# Combination of novel intravesical xenogeneic urothelial cell immunotherapy and chemotherapy enhances anti-tumor efficacy in preclinical murine bladder tumor models

**DOI:** 10.1007/s00262-020-02775-6

**Published:** 2020-11-06

**Authors:** Chi-Ping Huang, Chun-Chie Wu, Chih-Rong Shyr

**Affiliations:** 1 Division of Urology, China Medical University and Hospital, Taiwan, Republic of China; 2 Department of Medical Laboratory Science and Biotechnology, Sex Hormone Research Center, China Medical University and Hospital, Taiwan, Republic of China

**Keywords:** Xenotransplantation, Rejection, Immunity, Xenoantigen, Neoantigen

## Abstract

**Background:**

Immune checkpoint inhibitors induce robust and durable responses in advanced bladder cancer (BC), but only for a subset of patients. Xenovaccination has been proposed as an effective immunotherapeutic approach to induce anti-tumor immunity. Thus, we proposed a novel intravesical xenogeneic urothelial cell immunotherapy strategy to treat advanced BC based on the hypothesis that implanted xenogeneic urothelial cells not only provoke xeno-rejection immune responses but also elicit bystander anti-tumor immunity.

**Methods:**

Mouse advanced bladder cancer models were treated with vehicle control, intravesical xenogeneic urothelial cells, cisplatin + gemcitabine, or the combination and assessed for tumor responses to treatments. Tumors and spleens samples were collected for immunohistological staining, cellular and molecular analysis assessed by antibody staining, ELISA, cytotoxicity, and flow cytometry, respectively.

**Results:**

The combination treatment of xenogeneic urothelial cell immunotherapy with chemotherapy was more efficacious than either single therapy to extend survival time in MBT-2 graft bladder tumor model and to suppress tumor progression in murine carcinogen BBN-induced bladder tumor model. The single-cell immunotherapy and combined therapy increased more tumor-infiltrating immune cells in MBT-2 graft tumors compared to vehicle control and chemotherapy treatment groups. The activated T-cell proliferation, cytokine production, and cytotoxicity capacities were also higher in mice with xenogeneic urothelial cell immunotherapy and combination treatments.

**Conclusions:**

Our results suggest the potential for a novel xenogeneic urothelial cell-based immunotherapy alone and synergy with chemotherapy in the combination therapy. Therefore, our study supports developing xenogeneic urothelial cells as an immunotherapeutic agent in combination with chemotherapy for BC treatment.

**Electronic supplementary material:**

The online version of this article (10.1007/s00262-020-02775-6) contains supplementary material, which is available to authorized users.

## Introduction

Bladder cancer (BC) is a major disease with estimated 549,393 cases and 199,922 deaths annually worldwide [1]. Cisplatin-based systemic chemotherapy remains the mainstay of treatment in the first-line setting for advanced urothelial BC patients, with a median overall survival (OS) of 14–15 months and only approximately 5% of these patients have a 5-year survival [2, 3]. The therapeutic approach for advanced BC with antibodies against the immune checkpoint programmed death-ligand 1/programmed death-1 (PD-L1/PD-1) is effective, but only a subset of patients [4–6]. However, an update clinical study failed to show its survival benefit over chemotherapy in platinum-treated locally advanced or metastatic BC with PD-L1 expression ≥ 5%, further indicating the limited responses and effects of PD-L1/PD-1 checkpoint inhibitors and the need to develop novel strategies [7]. Intravesical bacillus Calmette–Guérin (BCG) immunotherapy using a live attenuated form of Mycobacterium bovis to treat high-risk superficial bladder carcinoma has been a standard therapeutic approach for decades to prevent recurrence by eliciting anti-bacteria inflammation that triggers a bystander anti-tumor immune responses [8, 9]. Based on the therapeutic concept of intravesical BCG immunotherapy, we have proposed a novel therapeutic hypothesis [10] and developed an innovative intravesical xenogeneic urothelial cell immunotherapy that could elicit the graft rejection mechanism to remove non-self xenogeneic urothelial cells, which could induce bystander anti-tumor immunity.

The direct intravesical transplantation of xenogeneic urothelial cell into bladders in clinic is a practice of xenogeneic cell therapy or cellular xenotransplantation [10, 11]. Xenotransplantation has long been proposed as a promising solution for donor shortage in transplantation, but it faces enormous immunological barriers from hyperacute, acute vascular rejection to delayed xenograft rejection sequentially [12]. However, xenogeneic cell therapy is more achievable as a clinical treatment, because no vascular tissues are involved and only cells are transplanted. Furthermore, xenogeneic vaccination which uses homologous antigens derived from different species (xenoantigens) were proposed as a cancer immunotherapy strategy by eliciting xenogeneic immune reaction to overcome immune ignorance and tolerance induced by tumor cells [13, 14].

We conjectured that intravesical implantation of xenogeneic urothelial cells would induce bystander anti-tumor effects and the combined xenogeneic urothelial cell immunotherapy and chemotherapy would most effectively treat advanced BC. In this study, we assessed the anti-tumor and immunologic effects of intravesical xenogeneic urothelial cell immunotherapy in single treatment and in combination with chemotherapy cytotoxic agents in two immunocompetent bladder tumor mouse models. We showed that xenogeneic urothelial cell immunotherapy alone extended survival and repressed tumor progression and in combination with chemotherapy cytotoxic agents can further promote longer survival and higher tumor reduction. Closer examination revealed that xenogeneic urothelial therapy promoted the infiltration and activation of T lymphocytes. Thus, we identified a role for xenogeneic urothelial cells in triggering rejection T-cell activation for anti-tumor activity.

## Materials and methods

### Mice

CH3/He and C57BL/6 mice were purchased from BioLASCO (Taipei, Taiwan). All mouse experiments and procedures were approved by the Institutional Animal Care and Use Committee (IACUC) review board at the China Medical University (Approval NO. CMUIACUC-2018-130).

### Xenogeneic urothelial cell isolation and culture from porcine source

Porcine urinary bladders for urothelial cell isolation were obtained from a local abattoir. Bladder tissues was dissected into 1- to 2-cm^2^ tissue pieces and treated with diapase II dissolved in HBSS (Gibco, Carlsbad, CA, USA) to strip the urothelium. The stripped urothelium was minced into small pieces and incubated in a cell isolation solution with type VI collagenase (Worthington, Lakewood, NJ, USA) in Hanks’ balanced salt solution (100 U/ml) to disaggregate the cells. Porcine epithelial cells were isolated and grown in the DMEM/Ham’s F12 medium supplemented with antibiotics (penicillin 100U/mL, streptomycin 100 mg/mL, and amphotericin B 5 mg/mL) and 10% FBS following the previously reported mouse urothelial cell culture protocol [15]. Passage 2–10 cells were used in the experiments. Cells were tested negative for mycoplasma, bacteria, yeast, and fungi before use.

### Orthotopic mouse MBT-2- luc graft bladder tumor model

Orthotopic MBT-2-luc graft bladder tumor murine model was generated according to the procedures in the previous study [15]. MBT-2 cells are murine BC cells with epithelial characteristics established from the bladder tumor induced by the administration of FANFT (*N*-[4-(5-nitro-2-furyl)-2-thiazolyl] formamide) to C3H/He mice [16]. Tumor growth was monitored by bioluminescence imaging (BLI) every 7 days after tumor inoculation by Xenogen IVIS 200 (Xenogen, Alameda, CA, USA). The tumor-bearing mice were divided into four treatment groups: (i) vehicle control, (ii) xenogeneic urothelial cell: intravesical instillation of xenogeneic urothelial cells (1 × 10^6^ cells in normal saline), once a week, day 3 for 4 weeks, (iii) gemcitabine plus cisplatin (GC) chemotherapy: intraperitoneal injection (IP) of gemcitabine (6 mg/mouse, day 1 and cisplatin (0.12 mg/mouse, day 2) once a week, for 4 weeks, and (iv) combined treatment. Treatment procedures were performed using sterile techniques. The responses of mice are analyzed on BLI total flux intensity changes from the baseline. The progress free survival (PFS) is defined at the time over than 20% increase in BLI total flux intensity of tumors compared with baseline and OS is defined at the time of death or the humane endpoint). Tumors were harvested and weighed when mice died, reached human endpoint or at the end of experiments.

### Mixed lymphocyte proliferation assay

For carboxyfluorescein diacetate succinimidyl ester (CFDA-SE) proliferation assay, lymphocytes from spleens of MBT-2-luc tumor-bearing mice with different treatments were incubated for 15 min in the darkness with 5 μM CFDA-SE (Thermo Fisher Scientific, Waltham, MA, USA) in PBS and then washed. The assay was performed by co-culturing 1 × 10^5^ target xenogeneic urothelial cells or MBT-2-luc cells together with 5 × 10^5^ CFDA-SE-labeled lymphocytes from spleens (E/T ratio 5:1) for 2 days. The intensity of CFDA-SE fluorescence in lymphocytes was measured by FACSCalibur flow cytometer (BD Biosciences, San Jose, CA, USA) and analyzed with FlowJo Software.

### Cytotoxicity assay

Target xenogeneic urothelial cells or MBT-2-luc cells were labeled with CFDA-SE which were plated for 24 h and then co-cultured with effector lymphocytes isolated from the spleens of mice with different treatments at effector/target (E:T) ratio = 10. After 4 h incubation, effector cells were removed, the fluorescent intensity of remaining adherent CFDA-SE labeled targets cells was measured by a fluorimeter. The intensity of CFDA-SE-labeled target cells without co-culturing effector cells was set as the baseline. Relative cytotoxic activity of effector lymphocytes from mice of different treatment was calculated from triplicate samples as [(Baseline intensity—experimental intensity)/(Baseline intensity)] and expressed as a percentage.

### Immunohistochemistry

MBT-2-luc tumors removed from the mice of different treated groups were fixed in formalin and embedded in paraffin and paraffin sections were stained with antibodies for CD4(GTX85525, GeneTex, Irvine, CA, USA), CD8 (GTX53126, GeneTex), CD56 (108577-T08, Sino Biological, Wayne, PA, USA), CD68 (ab125212, Abcam, Cambridge, MA, USA), and myeloperoxidase (MPO) (ab9535, Abcam) by the standard manufacturer’s procedures using automated Leica Bond III-autostainer (Leica Biosystems, Wetzlar, Germany). Numeration of staining positive cells was performed in four random high-power fields (HPF) of the tumor sections × 400 magnification, and expressed as average cell number per field.

### TUNEL assay

DNA fragmentation in apoptotic cells was detected by terminal deoxynucleotidyl transferase (TdT)-mediated dUTP nick end labeling (TUNEL), following the manufacturer’s protocol. (TUNEL BrightGreen Apoptosis Detection Kit, Vazyme Biotec, Nanjing, Jiangsu, China). All images were obtained using a microscope (Nikon Eclipse 80i) with an attached CCD camera.

### IFN-γ quantification by ELISA

IFN-γ level in culture medium of effector lymphocytes isolated from the MBT-2-luc tumor-bearing mice of different treated groups, stimulated by the co-culture of target xenogeneic cells or MBT-2-luc cells for 2 days, was evaluated using an enzyme-linked immunosorbent assay kit (Biolegend, San Diego, CA, USA) according to the manufacturer's protocol. Effector cells with co-culture served as a baseline control. Relative IFN-γ activation of effector cells stimulated by co-cultured target cells was calculated as follows: ([IFN-γ] co-culture − [IFN-γ] baseline)/([IFN-γ] baseline) × 100.

## BBN-induced bladder tumor mode

For *N*-butyl-*N*-(4-hydroxybutyl)-nitrosamine (BBN)-induced bladder tumor formation, a 0.05% concentration of BBN (TCI America, Portland, OR, USA) was dissolved in drinking water, and BBN-containing water in a dark bottle was provided to 8–10 week old female C57BL/6 mice ad libitum for 10–20 weeks until hematuria score is over 2 + (Aution Sticks urine strip, Arkray, Nakagyō-ku, Kyoto, Japan) as a sign of bladder tumor formation. BBN-induce tumor-bearing mice were treated according to the experimental scheme in MBT-2-luc tumor-bearing mice. Pathologic evaluation was performed on hematoxylin/eosin-stained paraffin sections of bladders, defined as follows: hyperplasia, carcinoma in situ (CIS), and invasion.

### Statistical analysis

Statistical analysis was performed using PASW Statistics 18. Graphs represent mean values ± standard error of the mean. *P *values were calculated using Student’s *t *test for comparing two groups. Survival analysis was determined by the log-rank test. *P* < 0.05 was considered statistically significant.

## Results

### Intravesical xenogeneic urothelial cell immunotherapy and GC chemotherapy combined treatment had a synergistic anti-tumor effect in the orthotopic MBT-2-luc graft bladder tumor mouse model

**Fig. 1:**
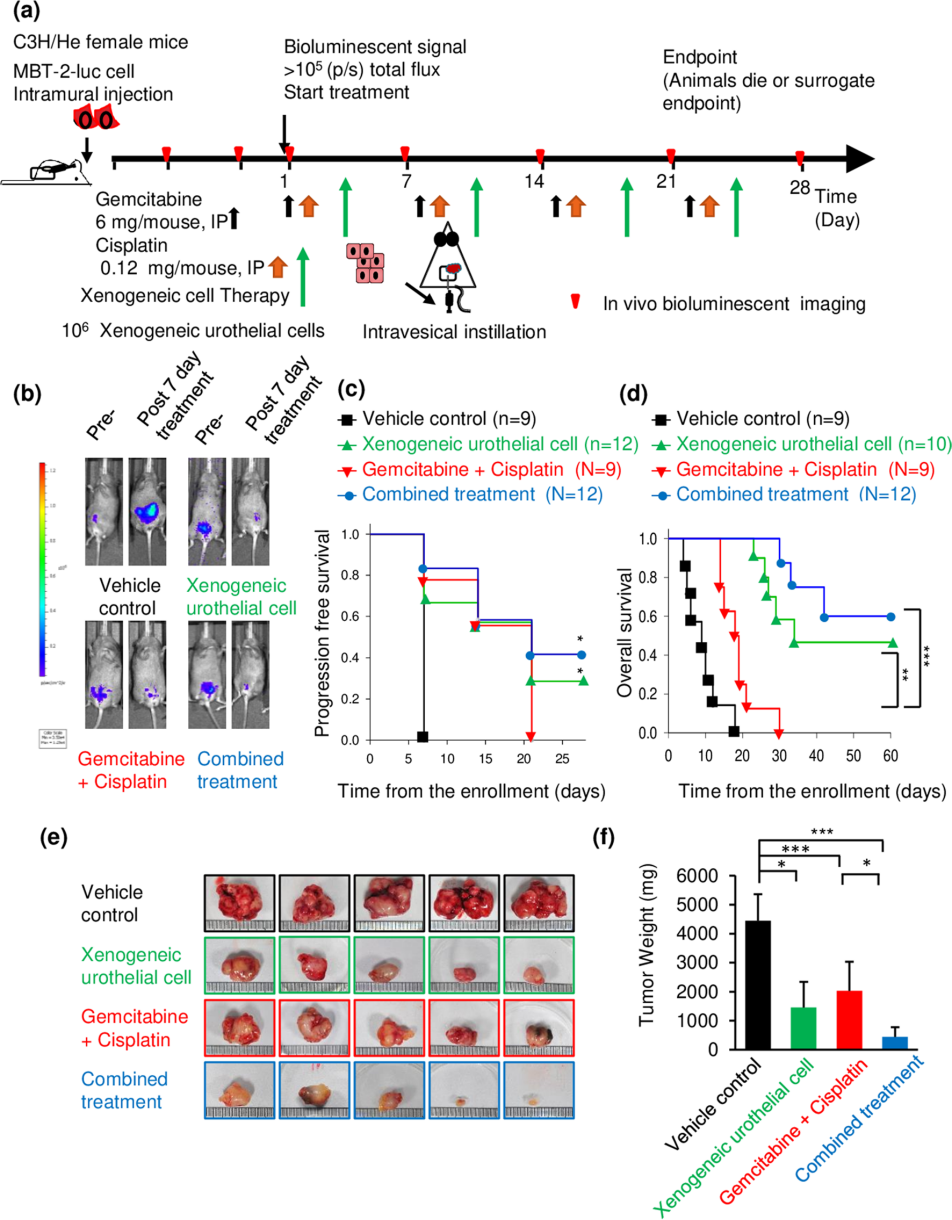
The anti-tumor effect of intravesical xenogeneic urothelial cell immunotherapy, GC chemotherapy and combined therapy in MBT-2-luc orthotopic graft bladder tumor model. **a** The tumor-bearing mice were enrolled when tumor bioluminescent signal reached 105 total plex and treated according to the treatment scheme. **b** The representative IVIS imagines of tumor-bearing mice before and after treatments were shown. **c** Kaplan–Meier progressive free survival curve (*p < 0.05 versus control) and **d** total survival curve. *p < 0.05; **p < 0.01; ***p < 0.001 versus chemotherapy treatment group. Kaplan–Meier survivor analysis with log-rank tests was performed by SigmaPlot 13. **e** Tumor morphology and **f** tumor weights of tumor-bearing mice with different treatments. Error bars represent SD. *p < 0.05; ***p < 0.001, by Student’s *t* test

Currently, the regime of gemcitabine plus cisplatin (GC) chemotherapy is a standard first-line therapy for patients with metastatic urothelial cancer of the bladder and urinary tract [17, 18]. However, most patients eventually experience disease progression or relapse after chemotherapy [19]. Thus, we first used the orthotopic graft urothelial bladder tumor mouse with MBT-2-luc cells to determine whether there are anti-tumor effects for xenogeneic urothelial cells as a single therapy as well as in combination with standard cytotoxic chemotherapy. We have successfully isolated and expanded xenogeneic urothelial cells from porcine bladders and demonstrated their ability to protect and repair damaged bladder urothelium [10]. The tumor-bearing mice treated with either xenogeneic urothelial cells, GC, or combination treatments all were shown effectively in suppressing tumor growth compared to the vehicle control group (Fig. 1b) and further prolonged progression free survival (median survival = 21, 16, and 34 days for xenogeneic urothelial cells, GC and combination treated groups, respectively, vs. 7 days for the vehicle control group) (Fig. 1c) and OS (median survival = 34, 18 and 67 days for xenogeneic urothelial cells, GC, and combination treated groups, respectively, vs. 9 days for the vehicle control group) (Fig. 1d). The cause of death in tumor-bearing mice was due to tumor progression that probably resulted in deteriorated kidney function and urinary congestion. And those treated with xenogeneic urothelial cells or combination showed about 30 and 40% durable response in PFS, respectively, and 45 and 60% in OS, respectively. At the end point, lower tumor weights were found in all treated group compared to control mice with average weights of 4443, 1456, 2027, and 444 mg for the vehicle control, xenogeneic urothelial cell, chemotherapy, and combination treated groups, respectively. Combined treatment showed lowest tumor weight among all groups (Fig. 1e, f). And the remnants of xenogeneic cells were detected by the presence of porcine mitochondrial cytochrome-b (Cyt-b) and D-Loop686 genes in tumors using real-time qPCR and the results showed that porcine mitochondrial DNA was still detectable 14 days after last injection (Supplementary Fig. S1). Although xenogeneic urothelial cell immunotherapy or gemcitabine and cisplatin chemotherapy alone all have anti-tumor activity, mice treated with both xenogeneic cell immunotherapy and GC chemotherapy exhibited a significant increase in tumor progressive free survival and total survival and combined treatment has highest survival.

### Intravesical xenogeneic urothelial cell immunotherapy, GC chemotherapy, and combined treatment decrease tumor cell proliferation and increased tumor cell apoptosis

**Fig. 2:**
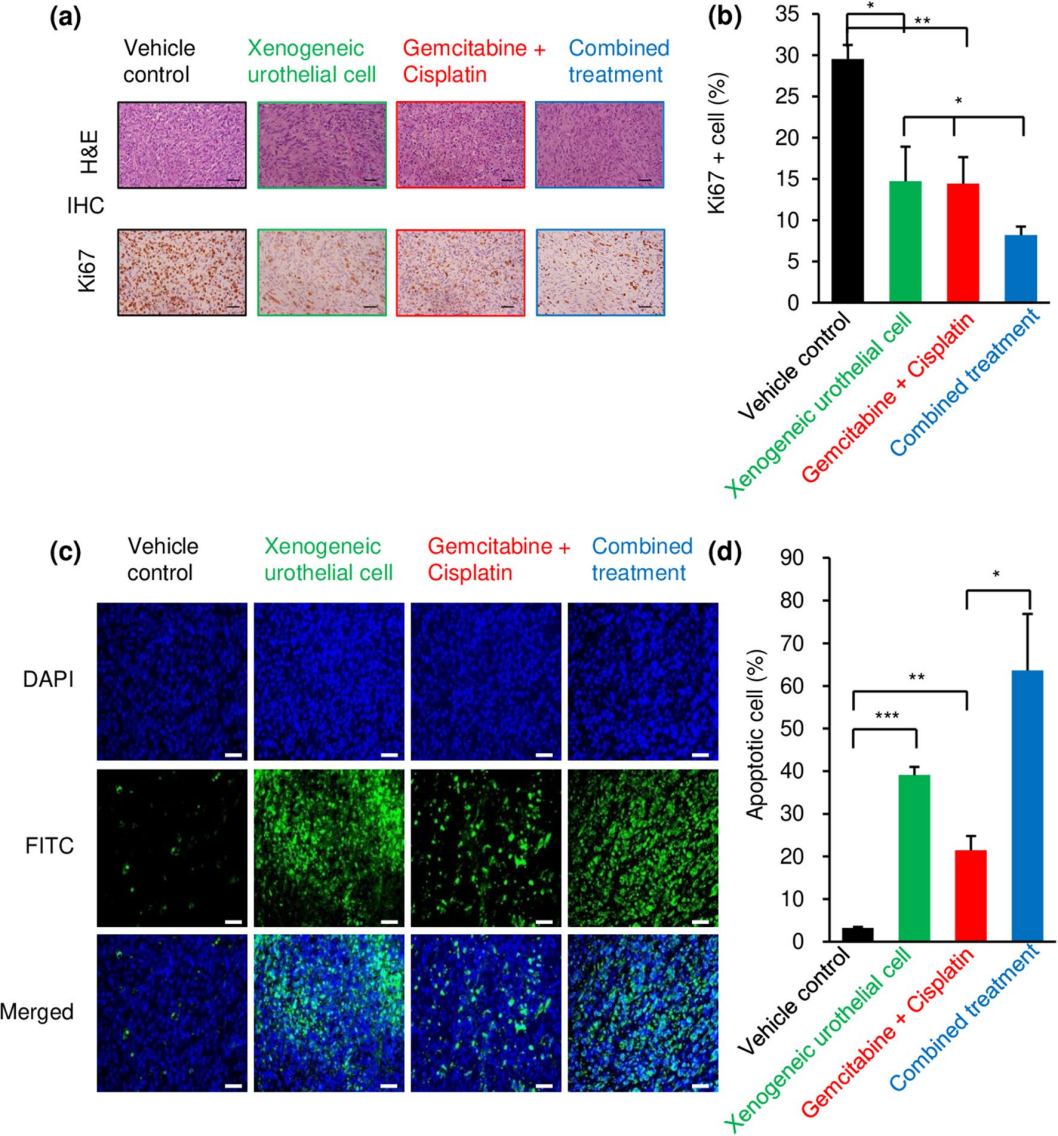
The effects of intravesical xenogeneic urothelial cell immunotherapy, GC chemotherapy, and combined therapy on tumor cell proliferation and survival in MBT-2-luc orthotopic graft urothelial bladder tumor model. **a** Tumors from mice of different treatment groups were harvested, processed, and sectioned for tumor cell Ki67 immunohistochemistry (IHC) and terminal deoxynucleotidyl transferase dUTP nick end labeling (TUNEL) assay. Tumor sections were evaluated for cell proliferation with Ki67 IHC staining. **b** Ki67-positive cells on tumor sections were counted and quantified from mice of three independent experiments. Scale bar, 100 μm. **c** Tumor sections were stained with TUNEL and observed under a fluorescent microscope. Scale bar, 50 μm. **d** The apoptotic nuclei are stained; green DAPI dye was used for nuclear staining (blue). TUNEL-positive cells on tumor sections were counted and quantified from mice of three independent experiments. Values are expressed as means ± standard deviation (SD) of the mean (*n* = 3). Error bars represent SD. *p < 0.05; **p < 0.01; ***p < 0.001, by Student’s *t* test

To determine the impact of xenogeneic urothelial cell immunotherapy on the anti-tumor effects, we first assessed the changes of tumor cells MBT-2-luc graft bladder tumor-bearing mice with the different treatments. During the period of responses, animals from each group were sacrificed and tumors were fixed and sectioned for Ki67 immunohistochemistry (IHC) staining to examine the cell proliferation and terminal deoxynucleotidyl transferase dUTP nick end labeling (TUNEL) assay for cell death. There were significantly fewer Ki67-positive tumor cells in the all treated groups compared with untreated control and the combination treatment had lowest number of proliferating cells (Fig. 2a, b). On the other hand, the TUNEL assay exhibited that apoptotic cells in tumor tissues were increased in all treatment group, but xenogeneic urothelial cell treatment showed higher number of apoptotic cells in the tumors than GC treatment. Additionally, a significant increase in the number of apoptotic cells was noted in the combined treatment group compared with each single treatment group (Fig. 2c, d).

### Intravesical xenogeneic urothelial cell immunotherapy, GC chemotherapy, and combined treatment enhanced immune cell infiltration in tumors

**Fig. 3:**
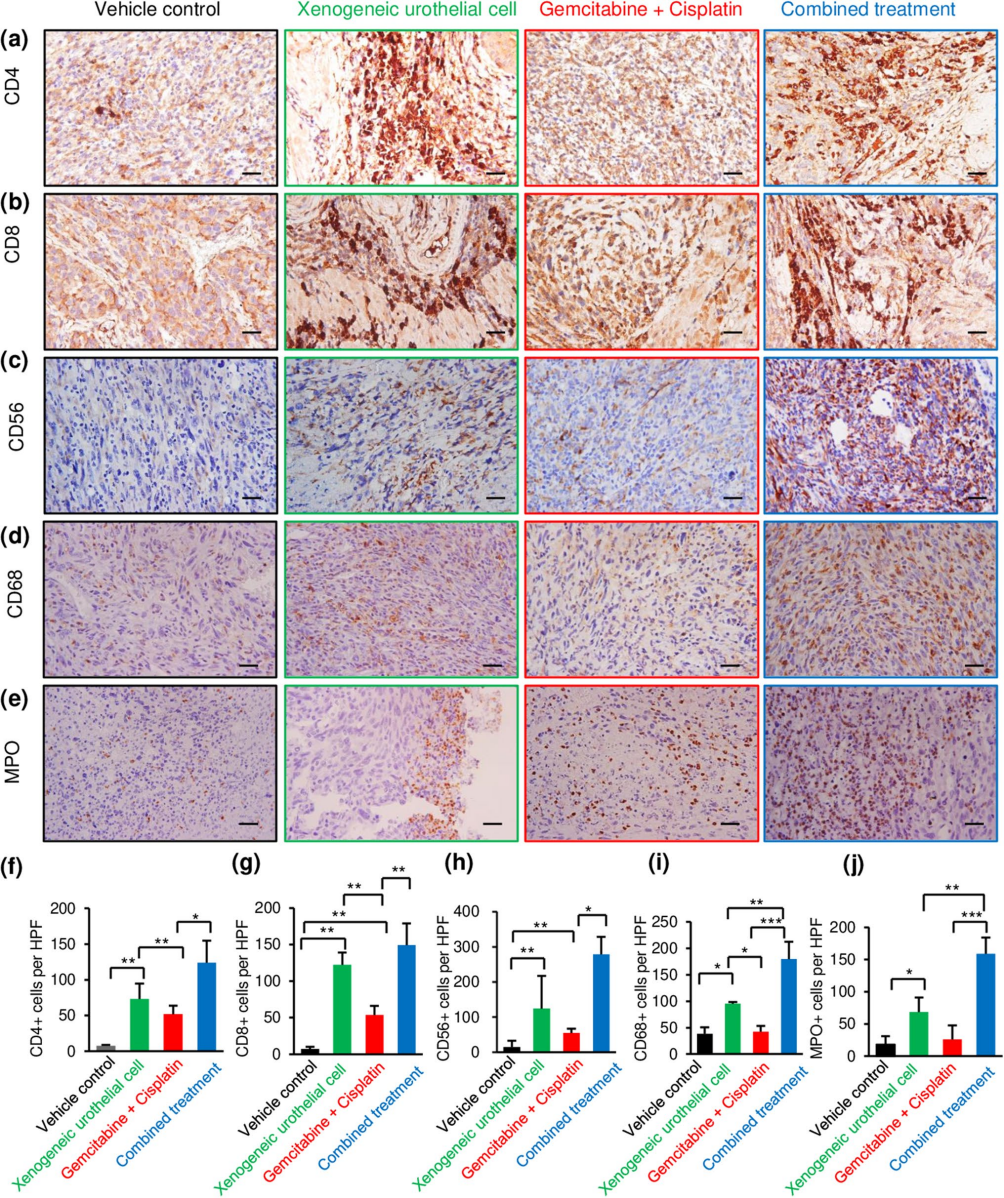
The changes in tumor-infiltrating lymphocytes and innate immune cells of MBT-2- luc tumor-bearing mice with different treatments. Tumors from mice given different treatments were analyzed by immunohistochemistry for lymphocyte and NK-cell infiltration. Representative images of **a** anti-CD4, **b** CD8, **c** CD56, **d** CD 68, and **e** MPO immunohistochemistry (IHC) staining on tumor sections. Scale bar, 100 μm. **f** Quantification of CD4 + T lymphocytes, **g** CD8 + T lymphocytes, **h** CD56 + NK cells, **i** CD 68 + monocytes, and **j** MPO + neutrophils in tumors for each treatment group by counting the positive cells in 4 HPFs that were randomly selected among three mice per group in each group, and data were expressed as the means ± SD. Error bars represent SD. *p < 0.05; **p < 0.01, by Student’s *t* test

To evaluate the impact of different treatments on intratumoral immune cell composition, we analyzed T-cell, NK-cell, monocyte, and neutrophil infiltration in tumors by IHC after treatments (Fig. 3a–e). Tumor T-cell infiltration (CD4 + and CD8 + effector T cells) was observed in both xenogeneic cell and GC treatment and the combined therapy further increased T-cell population, which reflects effective immunotherapy. Quantitative result showed that, compared to the vehicle-treated group tumors, xenogeneic urothelial cell-treated tumors from single and combined treatment groups were found to have a significant increase in both effector CD4 + T-cell (Fig. 3f) and CD8 + T-cell (Fig. 3g) infiltration in tumors and GC treatment also increased both effector T-cell infiltration. In the other hand, the increased NK-cell infiltration was only observed in tumors with xenogeneic urothelial cell single or combined treatment (Fig. 3h). Maker proteins for monocytes and neutrophils were also stained and results showed higher monocyte (Fig. 3i) and neutrophil (Fig. 3j) infiltration was also found in xenogeneic urothelial cell-treated group. To know whether the treatment was associated with tumor PD-L1 status, we performed PD-L1 IHC and found that tumors were stained weakly for PD-L1 expression in all groups and the expression level was not affected by different treatments (Supplementary Fig. S2).

### Intravesical xenogeneic urothelial cell immunotherapy, GC chemotherapy, and combined treatment activated immune responses

**Fig. 4:**
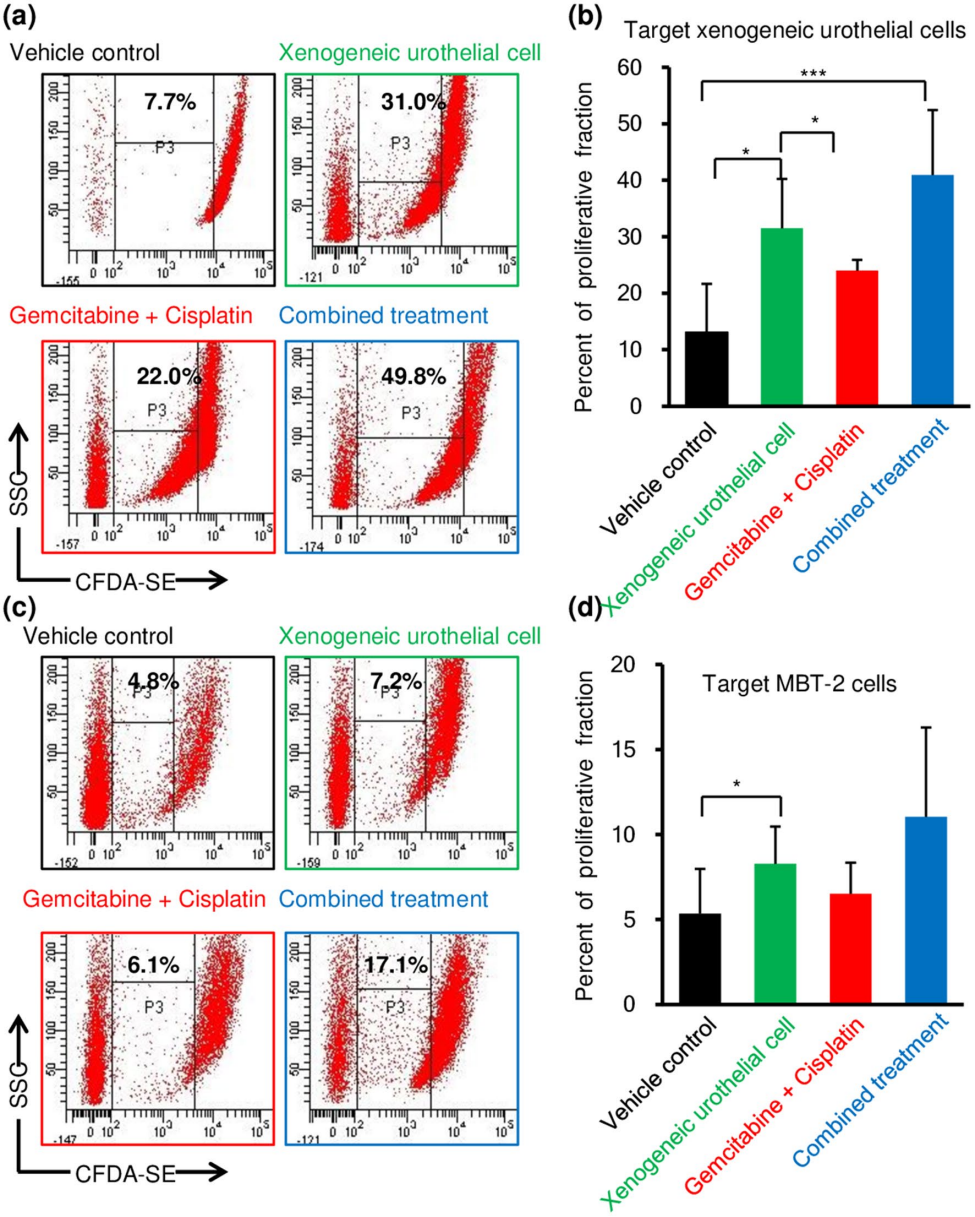
The immune activation on reactive T-cell proliferation in MBT-2-luc-tumor-bearing mice by different treatments. The mixed lymphocyte reaction was performed using lymphocytes isolated from the spleens of mice with different treatments. Lymphocytes were labeled with CFDA-SE and co-cultured with xenogeneic urothelial cells or MBT-2-luc cells. Proliferation of CFDA-SE‐labeled cells was measured by FACS analysis after 2 day culture. CFDA-SE‐labeled lymphocyte division was monitored with CFDA-SE labeling on cells to 5 × 10^5^ lymphocyte effector cells cultured with 1 × 10^5^ target xenogeneic cells or tumor cells. Flow cytometry analysis profiles of CFDA-SE-labeled lymphocytes after 2 day culture with **a** xenogeneic urothelial cells or **b** MBT-2-luc cells. **c** Percentage of proliferating lymphocytes responding to xenogeneic urothelial cells harvested on day 2 after treatment initiation. **d** Percentage of proliferating lymphocytes responding to MBT-2-luc cells. Error bars represent SD. *p < 0.05; ***p < 0.001, by Student’s *t* test

Since we hypothesized that xenogeneic cell immunotherapy could induce immune response due to xeno-rejection and collaterally increase anti-tumor immune responses, we tested whether T cells from mice treated with xenogeneic cells could exhibit enhanced proliferative responses when co-cultured with xenogeneic urothelial cells or bladder tumor cells by mixed lymphocyte reaction (MLR) using carboxyfluorescein diacetate succinimidyl ester (CFDA-SE)-based proliferation assay. To carry out this experiment, spleens were harvested and lymphocytes were isolated and labeled with CFDA-SE in PBS and then washed. The assay then was performed by culturing attached xenogeneic urothelial cells or tumor cells together with CFDA-SE-labeled lymphocytes isolated from the spleens of MBT-2-luc tumor-bearing mice with different treatments (effector/ target cells ratio 5:1) for 2 days. After that, lymphocytes were then harvested and analyzed by flow cytometry analysis to measure the intensity of CFDA-SE fluorescence in lymphocytes for measuring the proliferation of immune cells. We found that the CFDA-SE-labeled effector lymphocytes stimulated by co-culturing with xenogeneic urothelial cells showed that the proliferating proportion of lymphocytes (CFDA-SE low) from mice treated with xenogeneic urothelial cells was higher than that of mice treated with vehicle control, indicating that xenogeneic urothelial cell-treated mice developed immune response to xenogeneic cells (Fig. 4a, b). Moreover, CFDA-SE-labeled effector lymphocytes from mice treated xenogeneic urothelial cells also showed higher proliferating proportion when stimulated with MBT-2-luc bladder tumor cells (Fig. 4c, d). Increased lymphocyte proliferation was also found in combination treated mice, indicating that xenogeneic urothelial cell treatment induces immune responses in tumor-bearing mice to both implanting xenogeneic urothelial cells and tumor cells (Fig. 4a–d).

### Intravesical xenogeneic urothelial cell immunotherapy, GC chemotherapy, and combined treatment increased effector immune cell functions

**Fig. 5:**
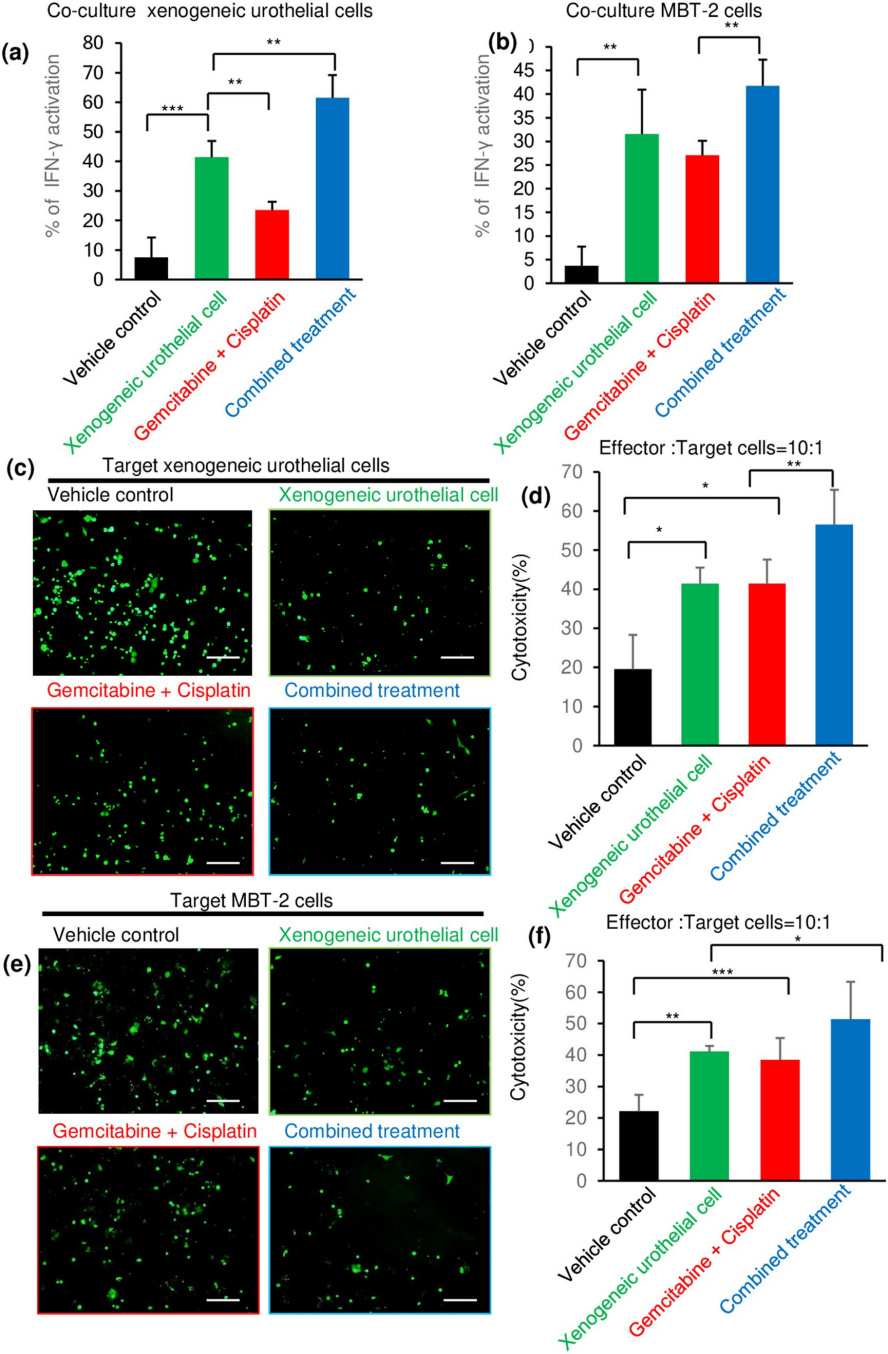
The stimulatory activity on reactive T-cell cytokine production and cytotoxicity in MBT-2-lu-tumor-bearing mice by different treatments. IFNγ level in supernatants collected after 2 days of co-culture of lymphocytes isolated from spleens of mice with different treatments with xenogeneic urothelial cells or MBT-2-luc cells was measured by ELISA. The supernatants of lymphocytes culture alone were used as the baseline control. Relative stimulatory activity of **a** xenogeneic urothelial cells and **b** MBT-2-luc on IFNγ production by lymphocytes from mice of different treatment group was calculated as the following: ([IFNγ]co-cultured—[IFNγ baseline)/ ([IFNγ ]baseline). IFNγ level in the supernatant of lymphocytes culture alone was used as the baseline. Effector lymphocytes (1 × 10^6^) from spleens of mice of different treatment groups were added into the plate seeded with 1 × 10^5^/well of target CFDA-SE-labeled effector xenogeneic urothelial cells (c&d) or MBT-2-luc (e&f) and co-cultured for 4 h. At the end of co-culture, suspension effector cells in the wells were washed out and the intensity of CFDA-SE-labeled target cells was measured. Representative fluorescent images of wells added with lymphocytes from mice with different treatments targeting **c** xenogeneic urothelial cells and **e** MBT-2-luc cells. The relative cytotoxic activity of lymphocytes was determined following the formula with measured relative fluorescence units (RFU) of each sample: (RFUexp-RFUctrl)/RFUctrl. The intensity of CFDA-SE-labeled target without adding lymphocytes was set as controls. Quantitation of effector lymphocyte cytotoxicity to target, **d** xenogeneic urothelial cells and **f** MBT-2-luc cells. Error bars represent SD. *p < 0.05; **p < 0.01; ***p < 0.001, by Student’s *t* test. Scale bar, 10 μm

We next evaluated the production of effector cytokine, interferon γ (IFNγ), in immune cells, which play an essential role in anti-tumor immunity [20]. Lymphocytes isolated from the spleens of tumor-bearing mice with different treatments were co-cultured with either xenogeneic urothelial cells or MBT-2-luc cells, and co-cultured conditioned medium were collected assayed for IFNγ activation in by ELISA. IFNγ activation when co-cultured with xenogeneic urothelial cells was higher in lymphocytes from xenogeneic urothelial cell-treated mice in both monotherapy and combined therapy groups (Fig. 5a). When co-cultured with MBT-2-luc cells, IFNγ activation was also higher in lymphocytes from xenogeneic urothelial cell-treated mice in both monotherapy and combined therapy groups (Fig. 5b). The increase in effector cytokine production by treatments correlated with the changes in effector cell proliferation, indicating that the implantation of xenogeneic urothelial cells regulates effector cell function. In addition, higher IFNγ activation reacting to co-culture of both xenogeneic urothelial cells and MBT-2-luc cells was also observed in lymphocytes from GC-treated mice, but its IFNγ activation reacting to co-cultured xenogeneic urothelial cells is less than the activation of the lymphocytes from xenogeneic urothelial cell-treated mice (Fig. 5a, b). Moreover, since CD8 + T cells’ cytotoxic activity is responsible for rejection the anti-tumor effects of cancer immunotherapy, we therefore asked whether xenogeneic urothelial cell immunotherapy could affect T cells’ cytotoxic activity function by determining immune effector cell-mediated target cell cytotoxicity. To examine the T-cell cytotoxic activity, we used lymphocytes isolated from spleens of mice with different treatment as effector cells. Using xenogeneic urothelial cells or MBT-2-luc cells as target cells, these cells were labeled with CFDA-SE and then in co-culture with effector cells isolated from spleens of mice of different treatment groups. After incubation, the cells were washed and the fluorescent intensity of remained CFDA-SE-labeled cells was measured. The highest cytotoxic activity (57%) to target xenogeneic urothelial cells was noted in the group of mice receiving combined treatment (Fig. 5c, d). Xenogeneic urothelial cell treatment had significant activation of cytotoxic cells and GC treatment also increased the cytotoxicity to target xenogeneic urothelial cells. When targeted on MBT-2-luc cells, the effectors cells from xenogeneic urothelial cell treatment and GC treatment mice increased the cytotoxicity to target MBT-2-luc cells and the cells from combination treated mice demonstrated more pronounced anti-MBT-2-luc cell cytotoxicity than the effector cells isolated from vehicle-treated mice (Fig. 5e, f).

### Intravesical xenogeneic urothelial cell immunotherapy and GC chemotherapy combined treatment synergistically delay tumor progression in the BBN-induced bladder tumor mouse model

**Fig. 6:**
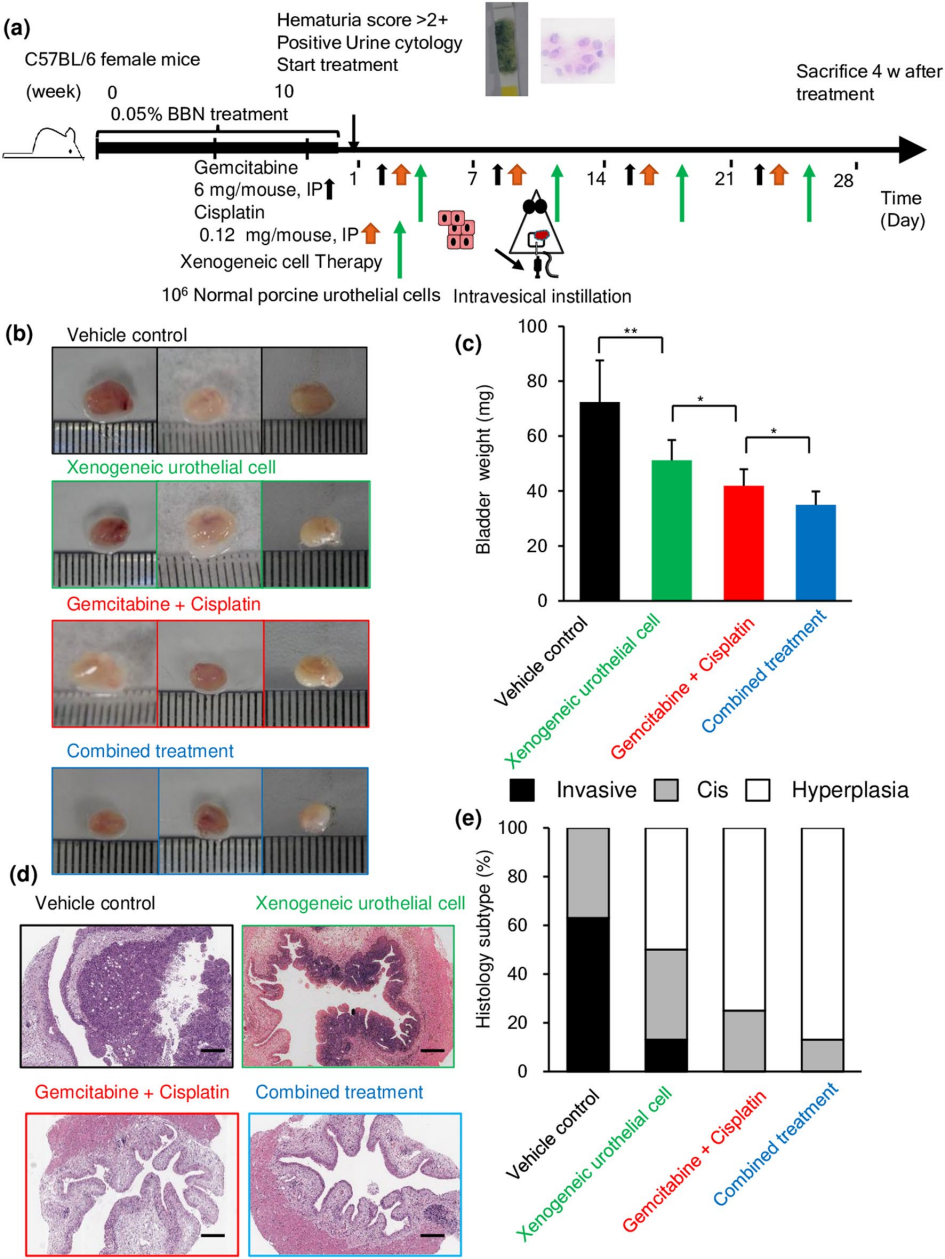
The anti-tumor effect of intravesical xenogeneic urothelial cell immunotherapy, GC chemotherapy, and combined therapy in the BBN-induced bladder tumor mouse model. **a** Tumor-bearing mice were treated according to the experimental scheme. **b** Representative photographs of the gross tumors from mice with different treatments. **c** Tumor weights of different treatment groups. **d** Representative HE images of tumors of different treatment groups. **e** Histograms of the pathological analysis for the proportions of hyperplastic and neoplastic changes in bladder HE section of mice with different treatments. Values are shown as means ± SD. Error bars represent SD. *p < 0.05; **p < 0.01 by Student’s *t* test. Scale bar, 250 μm

To test the anti-tumor effects of intravesical xenogeneic urothelial cell therapy in combination with chemotherapeutic agents in delaying tumor progression, we used the mouse BBN-induced tumor model, which simulates the human bladder tumorigenesis from hyperplasia, carcinoma in situ (CIS) to invasive carcinoma [21]. Our results demonstrated that intravesical xenogeneic urothelial cell immunotherapy alone or in combination of chemotherapy reduced the tumor weight and delayed tumor progression (Fig. 6b, c). When xenogeneic urothelial cells were used as monotherapy, there was significant reduction in tumor weight compared with vehicle-treated mice. GC treatment also repressed tumor progression by decreasing tumor weight. In addition, the combined treatment achieved highest decrease in tumor weight. Consistently with this macroscopic difference, histopathological analysis also showed significantly lower proportion of mice progressed to invasive carcinoma in the xenogeneic cell and GC-treated group and only a small portion of CIS (13%) and most hyperplasia were observed in combination treated mice (Fig. 6d, e).

## Discussion

In this study, we evaluated the therapeutic effects of xenogeneic urothelial cells as a monotherapy and the combined therapy with standard of care GC chemotherapy for advanced BC in two bladder tumor murine models. And we observed that intravesical xenogeneic urothelial cells could extend survival and suppress tumor progression in monotherapy and higher anti-tumor activity in the combined treatment. Thus, the data presented here demonstrate that the anti-tumor effects of xenogeneic urothelial cell immunotherapy and GC chemotherapy in combination enhanced anti-tumor activity over either treatment alone in two preclinical tumor murine models.

Tumor cells have many strategies to evade immunity either by downregulating the expression of tumor-specific or tumor-associated antigens on the cell surface, or inducing cells in the tumor microenvironment such as myeloid-derived suppressor cells or T regulatory cells to release cytokines, such as transforming growth factor beta (TGF-β), that suppress immune responses while promoting tumor cell proliferation and survival [22]. Our xenogeneic cell immunotherapy is able to activate a variety of innate and adaptive cytokines and immune cells, causing increased immune cell infiltration, cytokine production antigen spreading to eliminate tumor cells like a combination immunotherapy that is explored an approach to improving the efficacy of cancer immunotherapy [23]. Additionally, it is proposed that there is tremendous potential for synergistic combinations of immunotherapy agents and for combining immunotherapy agents with conventional cancer treatments like chemotherapy as a promising strategy to eradicate tumors [24]. Chemotherapy has been considered to be immune suppressive by repressing bone marrow function, but it is now accepted that certain chemotherapies can augment tumor immunity by inducing immunogenic cell death, releasing tumor antigens, and disrupting strategies that tumors use to evade immune recognition [25, 26].

In ex vivo studies, the result revealed that intravesical administration of xenogeneic urothelial cells could trigger T-cell activation to xenogeneic urothelial cells in cell proliferation and cytotoxicity as well as cytokine stimulation of IFNγ, which enable T-cell responses to reject tumors [27]. Furthermore, we also observed that IFNγ activation and cytotoxic activity of lymphocytes isolated from GC-treated mice not only reacted to graft MBT-2-luc cells but also to xenogeneic urothelial cells, suggesting that GC chemotherapy worked as cytotoxic agents and immune boosters to trigger anti-tumor immunity to reject tumor cells, which increased the body immunological rejection capacity to react to xenogeneic urothelial cells. Activation of Natural killer (NK) cells through cytokine production and/or direct or antibody-dependent cytotoxic lysis has been implicated in xenograft rejection in rodent and pig-to-nonhuman primate models [28, 29]. NK cells are also shown to act as immune effectors with their ability to “spontaneously” kill tumor cells for tumor surveillance and control [30]. Interleukin-2-(IL-2) activated tumor-infiltrating NK cells were demonstrated to have anti-tumor activity to shrink tumors in the murine tumor model [31]. Both innate and adaptive immunity induced by xenogeneic cells could all induce bystander immune action to anti-tumor immunity.

In xenotransplantation, activated T cells recognize xenoantigens through pre-existing xenoreactive T cells or indirect fashion that pro-inflammatory T cells reacting to xenoantigens indirectly by following recognition of xenogeneic peptides presented on recipient’s antigen‐presenting cells in an autologous MHC‐restricted fashion [32]. Tumors accumulate mutations through tumor development and these tumor-specific somatic mutations derived neoantigens [33]. These neoantigens recognized by activated anti-tumor T-cell repertoire have been shown to correlate with response to PD-L1/PD-1 antibodies in a range of advanced solid tumors, including advanced BC carcinoma and could be explored for novel immunotherapy approaches [34]. Since the rejection and anti-tumor immunity in humans share similarities, associating with the presence of activated T cells targeting xenoantigens or neoantigens respectively, xenogeneic cells with xenoantigens could in a bystander manner expand pre-existing neoantigen-specific T-cell populations, indicating the possibility of antigen spreading. The antigen spreading of an anti-tumor T-cell response to truly tumor-specific antigens has been proposed to contribute decisively to tumor regression [35]. We postulated that due to molecular mimicry, neoantigens share homology to xenoantigens. For example, TP53, whose mutations present in half of BC, differs in several amino acids cross-species (Supplementary Fig. S3), and thus, xenogeneic TP53 peptides of xenogeneic cells could be recognized by the host T-cell receptor (TCR) repertoire and serve as a partial surrogate for spreading differential neoantigen immunogenicity, leading to “bystander activation” of anti-tumor activity. Xenoantigens are immunogenic, but preserving an optimal homology range with host self proteins, and thus, xenogeneic vaccination has been tested in preclinical mouse models and several clinical studies. In mouse melanoma model, immunization with human tyrosinase-related protein 1 (hTRP1) (or gp75) elicited antibody or cytotoxic T-cell responses to gp75 and immunized mice rejected metastatic melanomas and developed patchy depigmentation in their coats [36]. Xenogeneic DNA immunization with xenogeneic human TRP-2 (hTRP2) DNA immunization prevented local recurrence and the development of metastases in a mouse model of minimal residual melanoma by a mechanism requiring CD4( +) and CD8( +) T cells [37]. Xenogeneic human tyrosinase plasmid DNA vaccination of dogs with advanced malignant melanoma showed potentially therapeutic activity [38]. In human clinical trials on malignant melanoma patients, DNA vaccines encoding xenogeneic melanosomal antigens (tyrosinase, gp100) induced CD8( +) T-cell responses [39] and epitope spreading of CD8 + T-cell response was observed [40].

Our novel intravesical xenogeneic urothelial immunotherapy is a local therapy with intravesical delivery of cells which enables local administration with minimal systemic exposure and efficient route to tumor sites, reducing the risk of potential severe immune reactions of systemic immunotherapy [41]. As a result, it could be applied in primary bladder tumors of locally advanced and metastatic BCs in monotherapy or combination with chemotherapy and even extended to non-muscle invasive BC and there has been a trial for patients with high-risk non-muscle invasive bladder cancer (NMIBC) unresponsive to bacillus Calmette–Guérin (BCG) treated with PD-1 inhibitors [42]. Yet, the risk of xenozoonotic (cross-species) infections such as porcine endogenous retroviruses poses another concern for the successful use of xenogeneic cell therapy in treating human diseases. However, the mitigation of xenozoonotic risks can be achieved through the use of donor pigs from designated pathogen-free (DPF) herds [43] and precisely removing porcine endogenous retrovirus genes anywhere in the genome of pigs with CRISPR/Cas9 genome editing technology [44] to reduce the risk of xenozoonotic infection for more possible clinical uses.

In conclusion, using two immunocompetent bladder tumor mouse models that recapitulates immune-tumor interaction to test our novel xenogeneic urothelial cell immunotherapy strategy, significant anti-tumor efficacy has been shown that therapeutic intravesical administration of xenogeneic urothelial cells facilitated T-cell activation and provoked vigorous anti-tumor immunity in monotherapy and had higher activity in combined treatment with standard of care chemotherapy. Thus, our preclinical study supports its evaluation for the treatment of BC in humans.

### Electronic supplementary material

Below is the link to the electronic supplementary material.

Supplementary file1 (PDF 150 KB)

Supplementary file2 (PDF 277 KB)

## Abbreviations

BBN: *N*-Butyl-*N*-(4-hydroxybutyl)-nitrosamine
BC: Bladder cancer
BCG: Bacillus Calmette–Guérin
BLI: Bioluminescence imaging
CFDA-SE: Carboxyfluorescein diacetate succinimidyl ester
CIS: Carcinoma in situ
GC: Gemcitabine plus cisplatin
IFN: Interferon
IHC: Immunohistochemistry
MLR: Mixed lymphocyte reaction
OS: Overall survival
PD-1: Programmed death-1
PD- L1: Programmed death-ligand 1
PFS: Progress free survival
TUNEL: Terminal deoxynucleotidyl transferase (TdT)-mediated dUTP nick end labeling

## References

[1] Bray F, Ferlay J, Soerjomataram I, Siegel RL, Torre LA, Jemal A (2018). Global cancer statistics 2018: GLOBOCAN estimates of incidence and mortality worldwide for 36 cancers in 185 countries. CA Cancer J Clin.

[2] Sternberg CN, de Mulder PH, Schornagel JH, Theodore C, Fossa SD, van Oosterom AT, Witjes F, Spina M, van Groeningen CJ, de Balincourt C, Collette L (2001). Randomized phase III trial of high-dose-intensity methotrexate, vinblastine, doxorubicin, and cisplatin (MVAC) chemotherapy and recombinant human granulocyte colony-stimulating factor versus classic MVAC in advanced urothelial tract tumors: European Organization for Research and Treatment of Cancer Protocol no. 30924. J Clin Oncol.

[3] Stenzl A, Cowan NC, De Santis M, Kuczyk MA, Merseburger AS, Ribal MJ, Sherif A, Witjes JA (2011). Treatment of muscle-invasive and metastatic bladder cancer: update of the EAU Guidelines. Eur Urol.

[4] Bellmunt J, Powles T, Vogelzang NJ (2017). A review on the evolution of PD-1/PD-L1 immunotherapy for bladder cancer: the future is now. Cancer Treat Rev.

[5] Rosenberg JE, Hoffman-Censits J, Powles T, van der Heijden MS, Balar AV, Necchi A, Dawson N, O'Donnell PH, Balmanoukian A, Loriot Y, Srinivas S, Retz MM, Grivas P, Joseph RW, Galsky MD, Fleming MT, Petrylak DP, Perez-Gracia JL, Burris HA, Castellano D, Canil C, Bellmunt J, Bajorin D, Nickles D, Bourgon R, Frampton GM, Cui N, Mariathasan S, Abidoye O, Fine GD, Dreicer R (2016). Atezolizumab in patients with locally advanced and metastatic urothelial carcinoma who have progressed following treatment with platinum-based chemotherapy: a single-arm, multicentre, phase 2 trial. Lancet.

[6] Fan Z, Liang Y, Yang X, Li B, Cui L, Luo L, Jia Y, Wang Y, Niu H (2019). A meta-analysis of the efficacy and safety of PD-1/PD-L1 immune checkpoint inhibitors as treatments for metastatic bladder cancer. Onco Targets Ther.

[7] Powles T, Duran I, van der Heijden MS, Loriot Y, Vogelzang NJ, De Giorgi U, Oudard S, Retz MM, Castellano D, Bamias A, Flechon A, Gravis G, Hussain S, Takano T, Leng N, Kadel EE, Banchereau R, Hegde PS, Mariathasan S, Cui N, Shen X, Derleth CL, Green MC, Ravaud A (2018). Atezolizumab versus chemotherapy in patients with platinum-treated locally advanced or metastatic urothelial carcinoma (IMvigor211): a multicentre, open-label, phase 3 randomised controlled trial. Lancet.

[8] Alexandroff AB, Jackson AM, O'Donnell MA, James K (1999). BCG immunotherapy of bladder cancer: 20 years on. Lancet.

[9] Sylvester RJ, van der Meijden AP, Witjes JA, Kurth K (2005). Bacillus calmette-guerin versus chemotherapy for the intravesical treatment of patients with carcinoma in situ of the bladder: a meta-analysis of the published results of randomized clinical trials. J Urol.

[10] Huang CP, Chen CC, Shyr CR (2018). Xenogeneic cell therapy provides a novel potential therapeutic option for cancers by restoring tissue function, repairing cancer wound and reviving anti-tumor immune responses. Cancer Cell Int.

[11] Edge AS, Gosse ME, Dinsmore J (1998). Xenogeneic cell therapy: current progress and future developments in porcine cell transplantation. Cell Transplant.

[12] Sykes M, Sachs DH (2019). Transplanting organs from pigs to humans. J Sci Immunol.

[13] Perales MA, Blachere NE, Engelhorn ME, Ferrone CR, Gold JS, Gregor PD, Noffz G, Wolchok JD, Houghton AN (2002). Strategies to overcome immune ignorance and tolerance. Semin Cancer Biol.

[14] Strioga MM, Darinskas A, Pasukoniene V, Mlynska A, Ostapenko V, Schijns V (2014). Xenogeneic therapeutic cancer vaccines as breakers of immune tolerance for clinical application: to use or not to use?. Vaccine.

[15] Huang CP, Chen CC, Shyr CR (2017). The anti-tumor effect of intravesical administration of normal urothelial cells on bladder cancer. Cytotherapy.

[16] Mickey DD, Mickey GH, Murphy WM, Niell HB, Soloway MS (1982). In vitro characterization of four *N*-[4-(5-nitro-2-furyl)-2-thiazolyl] formamide (FANFT) induced mouse bladder tumors. J Urol.

[17] von der Maase H, Hansen SW, Roberts JT, Dogliotti L, Oliver T, Moore MJ, Bodrogi I, Albers P, Knuth A, Lippert CM, Kerbrat P, Sanchez Rovira P, Wersall P, Cleall SP, Roychowdhury DF, Tomlin I, Visseren-Grul CM, Conte PF (2000). Gemcitabine and cisplatin versus methotrexate, vinblastine, doxorubicin, and cisplatin in advanced or metastatic bladder cancer: results of a large, randomized, multinational, multicenter, phase III study. J Clin Oncol.

[18] von der Maase H, Sengelov L, Roberts JT, Ricci S, Dogliotti L, Oliver T, Moore MJ, Zimmermann A, Arning M (2005). Long-term survival results of a randomized trial comparing gemcitabine plus cisplatin, with methotrexate, vinblastine, doxorubicin, plus cisplatin in patients with bladder cancer. J Clin Oncol.

[19] Stenzl A, Cowan NC, De Santis M, Jakse G, Kuczyk MA, Merseburger AS, Ribal MJ, Sherif A, Witjes JA (2009). The updated EAU guidelines on muscle-invasive and metastatic bladder cancer. Eur Urol.

[20] Nakajima C, Uekusa Y, Iwasaki M, Yamaguchi N, Mukai T, Gao P, Tomura M, Ono S, Tsujimura T, Fujiwara H, Hamaoka T (2001). A role of interferon-gamma (IFN-gamma) in tumor immunity: T cells with the capacity to reject tumor cells are generated but fail to migrate to tumor sites in IFN-gamma-deficient mice. Cancer Res.

[21] Becci PJ, Thompson HJ, Strum JM, Brown CC, Sporn MB, Moon RC (1981). *N*-butyl-*N*-(4-hydroxybutyl)nitrosamine-induced urinary bladder cancer in C57BL/6 X DBA/2 F1 mice as a useful model for study of chemoprevention of cancer with retinoids. Cancer Res.

[22] Chen DS, Mellman I (2017). Elements of cancer immunity and the cancer-immune set point. Nature.

[23] Moynihan KD, Opel CF, Szeto GL, Tzeng A, Zhu EF, Engreitz JM, Williams RT, Rakhra K, Zhang MH, Rothschilds AM, Kumari S, Kelly RL, Kwan BH, Abraham W, Hu K, Mehta NK, Kauke MJ, Suh H, Cochran JR, Lauffenburger DA, Wittrup KD, Irvine DJ (2016). Eradication of large established tumors in mice by combination immunotherapy that engages innate and adaptive immune responses. Nat Med.

[24] Melero I, Berman DM, Aznar MA, Korman AJ, Perez Gracia JL, Haanen J (2015). Evolving synergistic combinations of targeted immunotherapies to combat cancer. Nat Rev Cancer.

[25] Chen G, Emens LA (2013). Chemoimmunotherapy: reengineering tumor immunity. Cancer Immunol Immunother.

[26] Emens LA, Middleton G (2015). The interplay of immunotherapy and chemotherapy: harnessing potential synergies. Cancer Immunol Res.

[27] Gao J, Shi LZ, Zhao H, Chen J, Xiong L, He Q, Chen T, Roszik J, Bernatchez C, Woodman SE, Chen PL, Hwu P, Allison JP, Futreal A, Wargo JA, Sharma P (2016). Loss of IFN-gamma pathway genes in tumor cells as a mechanism of resistance to anti-CTLA-4 therapy. Cell.

[28] Tonomura N, Shimizu A, Wang S, Yamada K, Tchipashvili V, Weir GC, Yang YG (2008). Pig islet xenograft rejection in a mouse model with an established human immune system. Xenotransplantation.

[29] Cardona K, Korbutt GS, Milas Z, Lyon J, Cano J, Jiang W, Bello-Laborn H, Hacquoil B, Strobert E, Gangappa S, Weber CJ, Pearson TC, Rajotte RV, Larsen CP (2006). Long-term survival of neonatal porcine islets in nonhuman primates by targeting costimulation pathways. Nat Med.

[30] Sentman CL, Barber MA, Barber A, Zhang T (2006). NK cell receptors as tools in cancer immunotherapy. Adv Cancer Res.

[31] Yang Q, Hokland ME, Bryant JL, Zhang Y, Nannmark U, Watkins SC, Goldfarb RH, Herberman RB, Basse PH (2003). Tumor-localization by adoptively transferred, interleukin-2-activated NK cells leads to destruction of well-established lung metastases. Int J Cancer.

[32] Buhler L, Illigens BM, Nadazdin O, Tena A, Lee S, Sachs DH, Cooper DK, Benichou G (2016). Persistence of indirect but not direct T cell xenoresponses in baboon recipients of pig cell and organ transplants. Am J Transplant.

[33] Lawrence MS, Stojanov P, Polak P, Kryukov GV, Cibulskis K, Sivachenko A, Carter SL, Stewart C, Mermel CH, Roberts SA, Kiezun A, Hammerman PS, McKenna A, Drier Y, Zou L, Ramos AH, Pugh TJ, Stransky N, Helman E, Kim J, Sougnez C, Ambrogio L, Nickerson E, Shefler E, Cortes ML, Auclair D, Saksena G, Voet D, Noble M, DiCara D, Lin P, Lichtenstein L, Heiman DI, Fennell T, Imielinski M, Hernandez B, Hodis E, Baca S, Dulak AM, Lohr J, Landau DA, Wu CJ, Melendez-Zajgla J, Hidalgo-Miranda A, Koren A, McCarroll SA, Mora J, Lee RS, Crompton B, Onofrio R, Parkin M, Winckler W, Ardlie K, Gabriel SB, Roberts CW, Biegel JA, Stegmaier K, Bass AJ, Garraway LA, Meyerson M, Golub TR, Gordenin DA, Sunyaev S, Lander ES, Getz G (2013). Mutational heterogeneity in cancer and the search for new cancer-associated genes. Nature.

[34] Sahin U, Derhovanessian E, Miller M, Kloke BP, Simon P, Lower M, Bukur V, Tadmor AD, Luxemburger U, Schrors B, Omokoko T, Vormehr M, Albrecht C, Paruzynski A, Kuhn AN, Buck J, Heesch S, Schreeb KH, Muller F, Ortseifer I, Vogler I, Godehardt E, Attig S, Rae R, Breitkreuz A, Tolliver C, Suchan M, Martic G, Hohberger A, Sorn P, Diekmann J, Ciesla J, Waksmann O, Bruck AK, Witt M, Zillgen M, Rothermel A, Kasemann B, Langer D, Bolte S, Diken M, Kreiter S, Nemecek R, Gebhardt C, Grabbe S, Holler C, Utikal J, Huber C, Loquai C, Tureci O (2017). Personalized RNA mutanome vaccines mobilize poly-specific therapeutic immunity against cancer. Nature.

[35] Hardwick N, Chain B (2011). Epitope spreading contributes to effective immunotherapy in metastatic melanoma patients. Immunotherapy.

[36] Naftzger C, Takechi Y, Kohda H, Hara I, Vijayasaradhi S, Houghton AN (1996). Immune response to a differentiation antigen induced by altered antigen: a study of tumor rejection and autoimmunity. Proc Natl Acad Sci USA.

[37] Hawkins WG, Gold JS, Blachere NE, Bowne WB, Hoos A, Lewis JJ, Houghton AN (2002). Xenogeneic DNA immunization in melanoma models for minimal residual disease. J Surg Res.

[38] Bergman PJ, McKnight J, Novosad A, Charney S, Farrelly J, Craft D, Wulderk M, Jeffers Y, Sadelain M, Hohenhaus AE, Segal N, Gregor P, Engelhorn M, Riviere I, Houghton AN, Wolchok JD (2003). Long-term survival of dogs with advanced malignant melanoma after DNA vaccination with xenogeneic human tyrosinase: a phase I trial. Clin Cancer Res.

[39] Wolchok JD, Yuan J, Houghton AN, Gallardo HF, Rasalan TS, Wang J, Zhang Y, Ranganathan R, Chapman PB, Krown SE, Livingston PO, Heywood M, Riviere I, Panageas KS, Terzulli SL, Perales MA (2007). Safety and immunogenicity of tyrosinase DNA vaccines in patients with melanoma. Mol Ther.

[40] Ginsberg BA, Gallardo HF, Rasalan TS, Adamow M, Mu Z, Tandon S, Bewkes BB, Roman RA, Chapman PB, Schwartz GK, Carvajal RD, Panageas KS, Terzulli SL, Houghton AN, Yuan JD, Wolchok JD (2010). Immunologic response to xenogeneic gp100 DNA in melanoma patients: comparison of particle-mediated epidermal delivery with intramuscular injection. Clin Cancer Res.

[41] Abdel-Wahab N, Shah M, Suarez-Almazor ME (2016). Adverse events associated with immune checkpoint blockade in patients with cancer: a systematic review of case reports. PLoS ONE.

[42] Balar AV, Kulkarni GS, Uchio EM, Boormans J, Mourey L, Krieger LEM, Singer EA, Bajorin DF, Kamat AM, Grivas P, Seo HK, Nishiyama H, Konety BR, Nam K, Kapadia E, Frenkl TL, Wit RD (2019). Keynote 057: phase II trial of Pembrolizumab (pembro) for patients (pts) with high-risk (HR) nonmuscle invasive bladder cancer (NMIBC) unresponsive to Bacillus Calmette-Guérin (BCG). J Clin Oncol.

[43] Spizzo T, Denner J, Gazda L, Martin M, Nathu D, Scobie L, Takeuchi Y (2016). First update of the International Xenotransplantation Association consensus statement on conditions for undertaking clinical trials of porcine islet products in type 1 diabetes—chapter 2a source pigs—preventing xenozoonoses. Xenotransplantation.

[44] Niu D, Wei HJ, Lin L, George H, Wang T, Lee IH, Zhao HY, Wang Y, Kan Y, Shrock E, Lesha E, Wang G, Luo Y, Qing Y, Jiao D, Zhao H, Zhou X, Wang S, Wei H, Guell M, Church GM, Yang L (2017). Inactivation of porcine endogenous retrovirus in pigs using CRISPR-Cas9. Science.

