# Supplementary material for: Combination of novel intravesical xenogeneic urothelial cell immunotherapy and chemotherapy enhances anti-tumor efficacy in preclinical murine bladder tumor models
Source: Cancer Immunol Immunother. 2020 Nov 6;70(5):1419–33. doi: 10.1007/s00262-020-02775-6 (PMC8053151; doi:10.1007/s00262-020-02775-6)
Supplement: Supplementary file 1 — Supplementary file1 (PDF 150 KB) [file 262_2020_2775_MOESM1_ESM.pdf]

Supplementary Figure Captions

Fig. S1 The presence of porcine mitochondrial DNAs in tumors from xenogeneic urothelial cell-treated mice at different time points after injection. DNA was isolated from tumors for real-time qPCR with primers specific for porcine mitochondrial cytochrome-b (Cyt-b) and D-Loop686. Threshold cycles (Ct) were determined and plotted for each gene. PUC: Porcine urothelial cell DNA

Fig. S2 PD-L1 expression on tumors of different treatment groups. Tumors were harvested and processed for IHC using anti-PD-L1 antibody to stain the expression of PD-L1 protein on tumor cells. Scale bar, 100 μm.

Fig. S3 The alignment of Mouse, Pig and Human P53 amino acid sequences was performed using the Uniprot 'Align' tool. An * (asterisk) indicates positions which have a single, fully conserved residue. A : (colon) indicates conservation between groups of strongly similar properties - scoring > 0.5 in the Gonnet PAM 250 matrix. A . (period) indicates conservation between groups of weakly similar properties - scoring =< 0.5 in the Gonnet PAM 250 matrix.
