# Supplementary figures and images for: Combination of novel intravesical xenogeneic urothelial cell immunotherapy and chemotherapy enhances anti-tumor efficacy in preclinical murine bladder tumor models
Source: Cancer Immunol Immunother. 2020 Nov 6;70(5):1419–33. doi: 10.1007/s00262-020-02775-6 (PMC8053151; doi:10.1007/s00262-020-02775-6)

Fig. S1

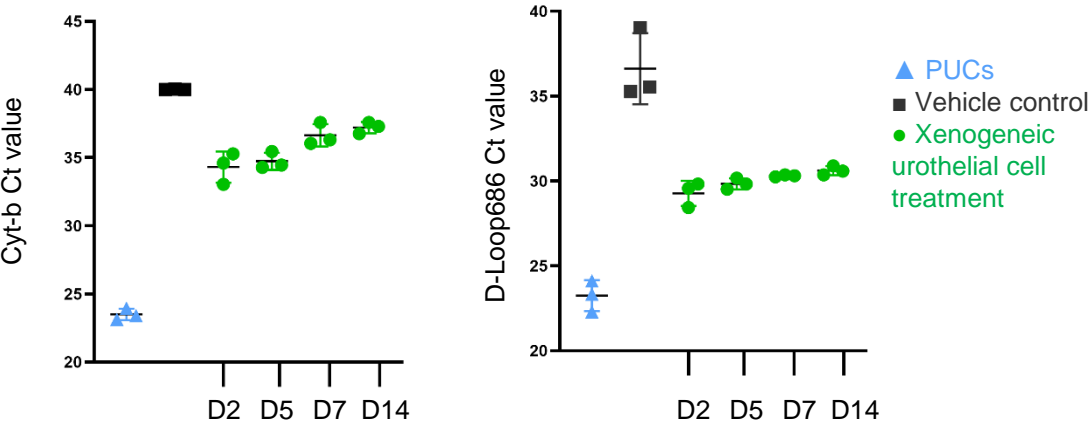

Fig. S2

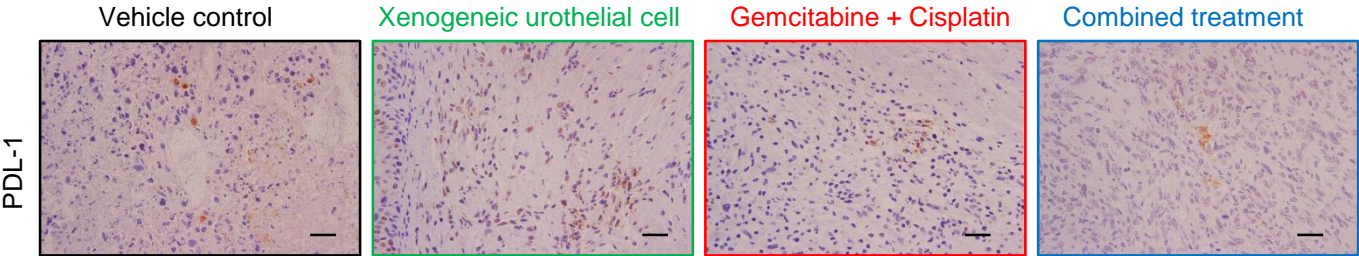

Fig. S3

[illegible]

Supplement: Supplementary file 2 — Supplementary file2 (PDF 277 KB) [file 262_2020_2775_MOESM2_ESM.pdf]
